# Durable protection against lethal Rift Valley fever hepatitis and encephalitis following low-dose ΔNSsΔNSm vaccination in mice

**DOI:** 10.1128/msphere.00894-25

**Published:** 2026-02-27

**Authors:** Karina Mueller Brown, Angel M. Kindsvogel, Lingqing Xu, Ashley M. Divens, Anita K. McElroy

**Affiliations:** 1 Division of Pediatric Infection Disease, Department of Pediatrics, School of Medicine, University of Pittsburgh 209879, Pittsburgh, Pennsylvania, USA; 2 Center for Vaccine Research, University of Pittsburgh 588296, Pittsburgh, Pennsylvania, USA; University of California Davis, Davis, California, USA

**Keywords:** Rift Valley fever virus, hepatitis, immunization, encephalitis, protection

## Abstract

Rift Valley fever virus (RVFV) is a zoonotic arbovirus that infects and causes disease in both humans and livestock. In humans, RVFV infection mostly results in febrile illness but has the potential to cause severe complications such as hepatitis and encephalitis. There are currently no licensed vaccines for human use; however, several candidates have been shown to be safe and immunogenic in pre-clinical studies. However, there remain gaps in our knowledge, including the duration of immunity and efficacy of protection from divergent disease manifestations over time. This pilot study investigated the duration of immunity following low-dose vaccination with live-attenuated RVFV ΔNSsΔNSm in two murine models with divergent RVF disease manifestations. Following percutaneous infection with wild-type (WT) RVFV, C57BL/6 mice readily succumb to fulminant hepatitis 3 to 5 days post-infection (dpi). In contrast, CC057/Unc mice from the Collaborative Cross Resource experience self-limiting liver disease and develop late-onset encephalitis. Following ΔNSsΔNSm vaccination, both humoral and cellular immunity decreased over time but were comparable between the two mouse strains at 1 and 3 months. Vaccinated mice were challenged with WT RVFV (ZH501) 6 months later, and 83% of C57BL/6 mice and 100% of CC057/Unc mice survived. Taken together, this study highlights the potential of ΔNSsΔNSm to provide durable protection against lethal RVF hepatitis and encephalitis.

**IMPORTANCE:**

Rift Valley fever virus (RVFV) causes both morbidity and mortality in endemic areas of Africa and the Middle East; however, no vaccines are available for humans. Pre-clinical studies have investigated the safety and immunogenicity of ΔNSsΔNSm vaccine (a live-attenuated version of RVFV that has two virulence factors deleted), but the duration of immunity and protection in the context of divergent RVF disease manifestations was unknown. This pilot study demonstrated that a single, low dose of ΔNSsΔNSm provided substantial protection to mice from RVF hepatitis and encephalitis 6 months post-vaccination. These pre-clinical data could support further development of a live-attenuated vaccine based on this platform for human use.

## OBSERVATION

Rift Valley fever virus (RVFV) is a zoonotic arbovirus endemic in Africa and the Arabian Peninsula infecting and causing disease in humans and livestock (1, 2). In humans, RVFV infection predominantly causes febrile illness but can cause serious complications such as ocular disease, hemorrhagic fever, hepatitis, and encephalitis (3). Previous studies have utilized murine models to understand the many facets of RVFV pathogenesis and investigate the potential preventative measures (1, 2, 4, 5). Several RVFV vaccine platforms have been tested to date in a variety of animal models (4). This includes the live-attenuated candidate ΔNSsΔNSm, which lacks the viral nonstructural proteins NSs and NSm. NSs is a strong antagonist of the innate immune response (6, 7), while NSm has anti-apoptotic functions and has been implicated in viral replication in mosquitos (8). Of note, the NSm open reading frame overlaps with that of the 78 kDa Gn/Gc protein, which has been postulated to play a role in mosquito infection (9). Vaccination with ΔNSs or ΔNSsΔNSm has been shown to be safe and immunogenic in several animal models (10–16). This pilot study utilized two murine models of divergent RVF disease. C57BL/6 mice succumb to lethal acute hepatic disease when challenged with WT RVFV (17). In contrast, CC057/Unc mice from the Collaborative Cross Resource display self-limiting liver disease when challenged with WT RVFV but subsequently develop and succumb to encephalitis (18). It was previously shown that C57BL/6 mice were protected 1 month post-ΔNSs vaccination or when receiving passively transferred serum from ΔNSs vaccinated animals prior to challenge (12). Protection following ΔNSsΔNSm vaccination has not yet been explored in C57BL/6 mice. CC057/Unc mice were protected 1 month post-ΔNSs or ΔNSsΔNSm vaccination or when passively transferred serum from ΔNSs vaccinated animals (11). However, long-term protection of either mouse strain following ΔNSsΔNSm vaccination has not yet been explored. Given limited resources, including the availability of CC057/Unc mice, preliminary data are needed to justify a larger, well-powered vaccine study. Thus, this pilot study aimed to investigate the duration of immunity and protection from a single, low-dose of ΔNSsΔNSm in both murine models over a 6-month period on a smaller scale.

## LOW-DOSE ΔNSsΔNSm VACCINATION ELICITS COMPARABLE IMMUNE RESPONSES IN C57BL/6 AND CC057/Unc MICE

**Fig 1:**
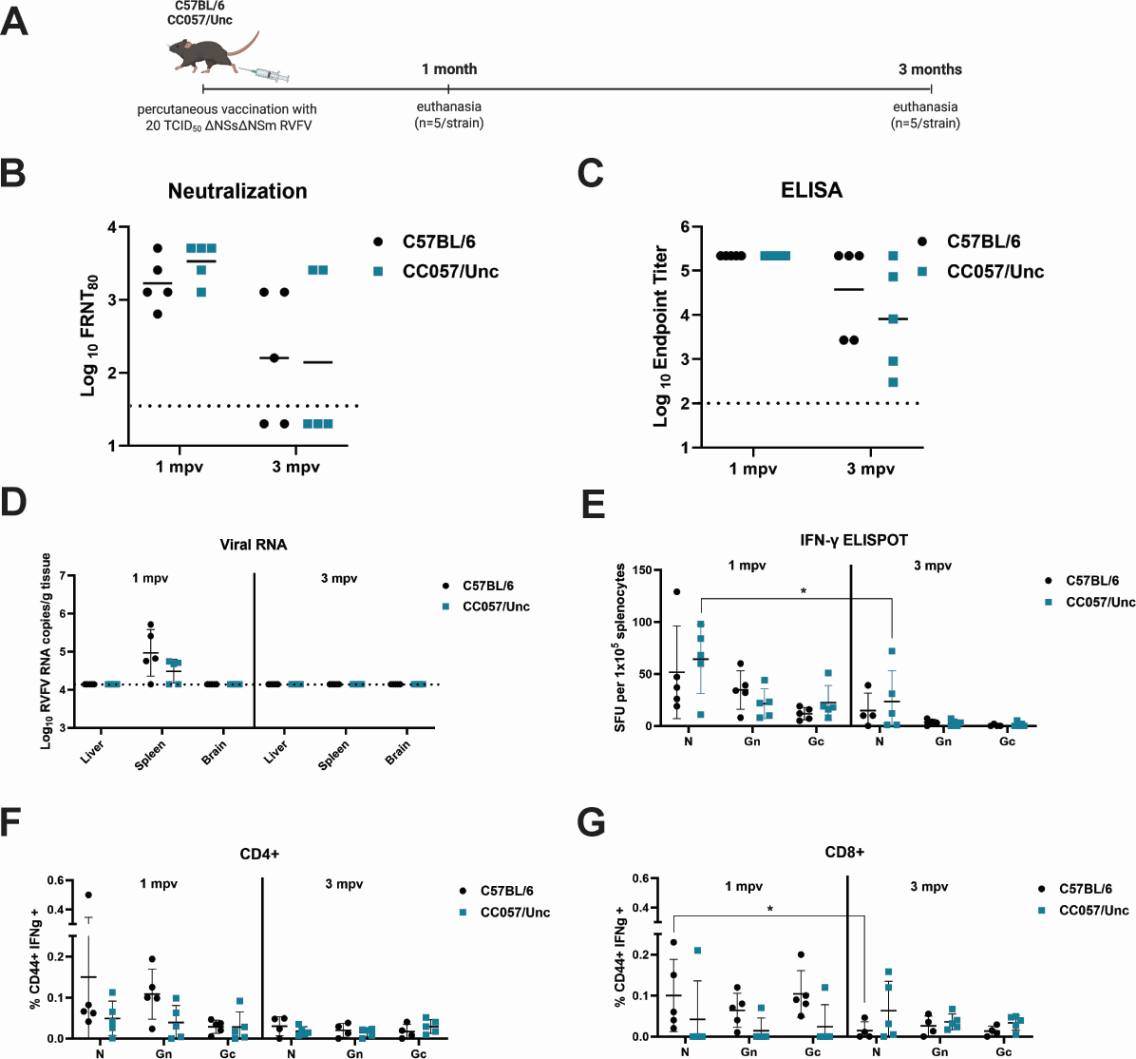
A single, low-dose vaccination with ΔNSsΔNSm elicits comparable immunity in C57BL/6 and CC057/Unc mice. (**A**) C57BL/6 or CC057/Unc mice (*n* = 10/strain) were vaccinated with 20 TCID_50_ ΔNSsΔNSm RVFV via foot pad injection. Mice were then either euthanized at 1 month (*n* = 5/strain) or 3 months (*n* = 5/strain) post-vaccination (mpv). The image was created using BioRender.com. (**B, C**) The endpoint titers of total RVFV-specific antibodies by neutralization or ELISA titer were measured at the time of euthanasia. The limit of detection (LOD) is depicted as a dotted line at 40 for focus reduction neutralization test (FRNT) and at 100 for ELISA. Negative values were plotted at 20 for FRNT_80_. (**D**) Terminal viral RNA loads in the liver, spleen, and brain were measured for RVFV L segment. The dotted line represents the LOD of the assay, and values below LOD are plotted at LOD (1.4 × 10^4^ copies/g). (**E**) The number of RVFV-specific T cells was assessed by IFN-γ ELISpot assay, expressed as the number of spot-forming units (SFUs) per 1 × 10^5^ splenocytes for RVFV N, Gn, and Gc. (**F, G**) The percentage of RVFV-specific CD4+ and CD8+ T cells was determined by flow cytometry by staining for CD44 and IFN-γ. Graphs depict the mean and standard deviation (SD) for linear data and the geometric mean for logarithmic data. The 3 mpv time point only has *n* = 4 C57BL/6 mice for the cellular immunity analysis (**E–G**). Statistical analysis was performed using two-way ANOVA by antibody titer or tissue viral load or viral protein and mouse strain (all not significant) or by antibody titer or tissue viral load or viral protein and time with *P < 0.05.

To compare the duration of humoral and cellular immunity, C57BL/6 and CC057/Unc mice (*n* = 10/strain) were vaccinated with a low-dose of ΔNSsΔNSm (20 TCID_50_) and timed euthanasia (*n* = 5/strain/time point) was performed at 1 and 3 months post-vaccination (mpv) (Fig. 1A). Humoral immunity decreased over time, independent of mouse strain, as measured by both neutralizing (Fig. 1B) and binding antibodies (Fig. 1C). Viral RNA was detectable in the spleen at 1 mpv but was undetectable in collected tissues by 3 mpv (Fig. 1D). Cellular immunity, as measured by virus-specific T cells, was tested via an *ex vivo* peptide stimulation assay using peptide pools that represent the following viral structural proteins: nucleoprotein (N) and glycoproteins (Gn and Gc) (Fig. 1E through G). Overall, there was a contraction of virus-specific T cells from 1 mpv to 3 mpv for both mouse strains. There were statistically significant differences for RVFV N-specific T cells in CC057/Unc mice by ELISpot assay (Fig. 1E) and RVFV N-specific CD8+ T cells in C57BL/6 mice by flow cytometric assay (Fig. 1G) between the 1 mpv and 3 mpv time point. There were no statistically significant differences between C57BL/6 and CC057/Unc mice across experiments (Fig. 1). In conclusion, although humoral and cellular immune responses decreased over time, they were still detectable by 3 mpv and were comparable between C57BL/6 and CC057/Unc mice.

## LOW-DOSE ΔNSsΔNSm VACCINATION PROVIDES DURABLE PROTECTION AGAINST LETHAL RVF HEPATITIS AND ENCEPHALITIS

**Fig 2:**
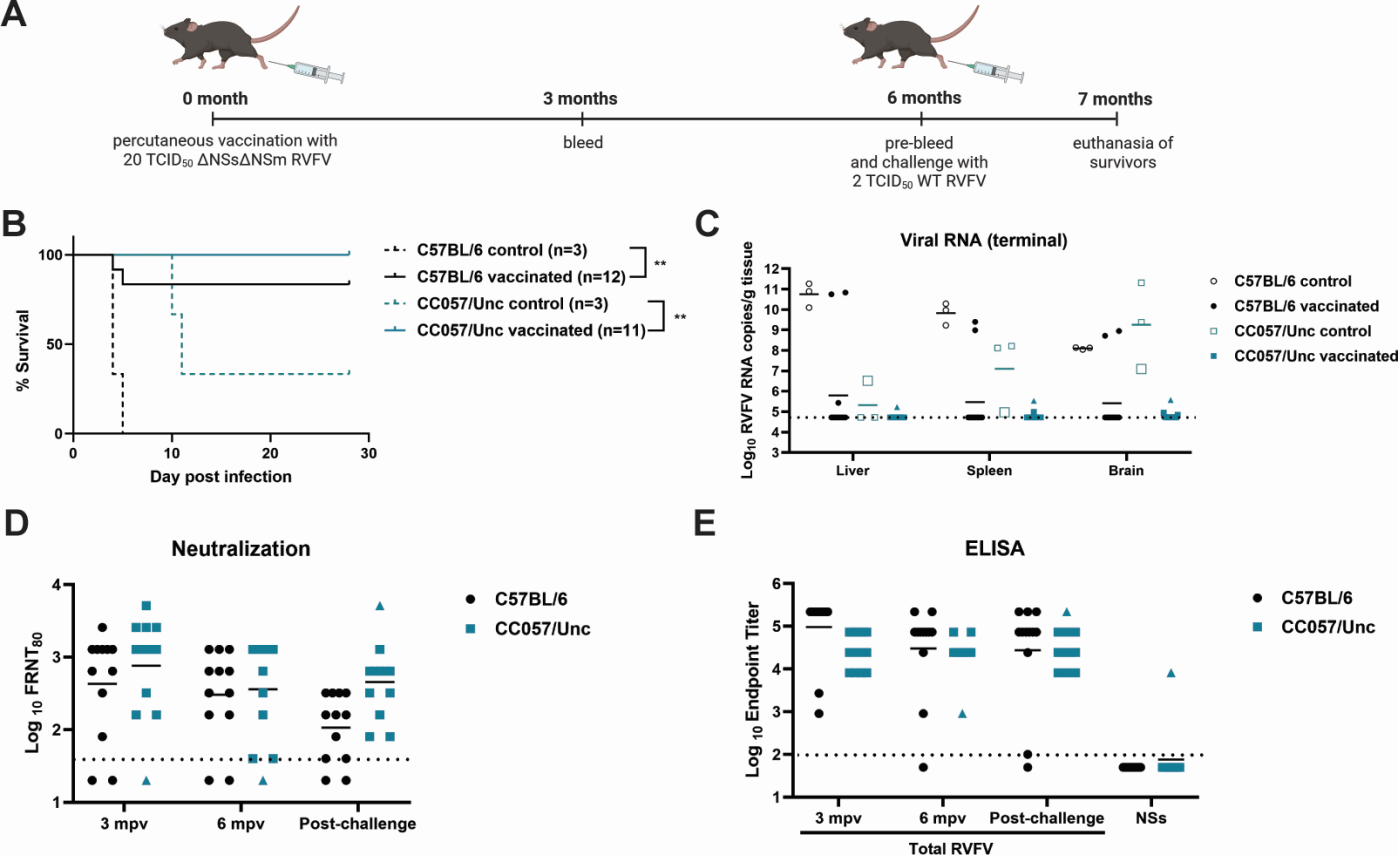
Protection of C57BL/6 and CC057/Unc mice from lethal RVF hepatitis and encephalitis at 6 months post-ΔNSsΔNSm vaccination. (**A**) C57BL/6 and CC057/Unc mice (*n* = 11–12/strain) were vaccinated with 20 TCID_50_ ΔNSsΔNSm RVFV via foot pad injection and bled at 3 and 6 months post-vaccination (mpv). The image was created using BioRender.com. Age-matched control animals (*n* = 3/strain) and vaccinated animals were challenged at 6 mpv with 2 TCID_50_ WT RVFV. (**B**) Animals were monitored for survival, and survivors were euthanized at 28 days post-challenge. Statistical analysis was performed using the Mantel-Cox test with **P < 0.01. (**C**) Terminal viral RNA loads in the liver, spleen, and brain were measured for RVFV L segment. The dotted line represents the LOD of the assay at 5 × 10^4^ copies/g, which is also where negative values are plotted. (**D, E**) The neutralization titer or endpoint titers of total RVFV-specific or RVFV NSs-specific antibodies by ELISA were measured at 3 mpv, 6 mpv, and at the time of euthanasia (post-challenge). The LOD is depicted as a dotted line at 40 for focus reduction neutralization test (FRNT) and at 100 for ELISA. Negative values were plotted at 20 for FRNT_80_ and 50 for ELISA. The larger open square in B represents the CC057/Unc control mouse survivor. The triangle symbol in C, D, and E represents an animal that had boosted antibodies post-challenge. Graphs depict the geometric mean.

To compare the duration of protection, C57BL/6 and CC057/Unc mice (*n* = 12/strain) were challenged with WT RVFV ZH501 (2 TCID_50_) 6 months post-ΔNSsΔNSm vaccination (20 TCID_50_) (Fig. 2). Of the vaccinated C57BL/6 mice, 83% (10/12) survived lethal WT challenge (Fig. 2B). These survivors had little to no detectable viral RNA in the liver, spleen, or brain (Fig. 2C) and did not boost in their humoral immune response post-challenge (Fig. 2D and E). The two vaccinated C57BL/6 mice that succumbed to RVF hepatitis showed high viral RNA titers in the collected tissues similar to the C57BL/6 control animals that received no vaccination and succumbed to RVF hepatitis at 4 and 5 dpi (Fig. 2B and C). Furthermore, these two animals had no detectable neutralizing antibodies and very low binding titers at the time of euthanasia (Fig. 2D and E), suggesting that low-dose vaccination was not sufficient to provide protection to all C57BL/6 mice. All vaccinated CC057/Unc mice survived WT RVFV challenge (Fig. 2B) and showed very low viral RNA in the tissues (Fig. 2C). Two of the CC057/Unc control mice succumbed to encephalitis at 10 and 11 dpi as reflected by high viral RNA titers in the brain (Fig. 2C). The surviving CC057/Unc control mouse lost 19% of its body weight by 17 dpi (20% is euthanasia criteria) but then started to regain weight and remained stable until the end of the study (Fig. S1). Importantly, this mouse had high viral RNA in the brain at the time of euthanasia, suggesting CNS disease (Fig. 2C, larger open square). Vaccinated CC057/Unc mice did not display increases in humoral immunity post-challenge (Fig. 2D and E) except for one animal (Fig. 2D and E, triangle symbol). This CC057/Unc mouse showed detectable neutralizing antibody only after WT challenge (Fig. 2D, triangle symbol) and had detectable anti-NSs binding antibodies (Fig. 2E), indicating that it had an acute WT infection. However, the animal did not experience any clinical signs of disease, as opposed to the surviving CC057/Unc control mouse, suggesting that vaccination provided protection from disease. Overall, low-dose ΔNSsΔNSm vaccination provided durable protection at 6 months in both mouse strains.

This pilot study aimed to investigate the duration of immunity and protection following a low-dose ΔNSsΔNSm vaccination in C57BL/6 and CC057/Unc mice. Both mouse strains had comparable immunogenicity by geometric mean titers, except for the two C57BL/6 mice that were not protected from WT challenge and had no detectable virus-specific neutralizing antibodies and lower ELISA titers at the time of challenge. Prior work has demonstrated an association between neutralizing titers and protection (12, 19, 20); hence, this finding was not unexpected. Both male and female mice were used in this study, and they were sex-matched based upon availability of the CC057/Unc mice, which produce small, male-dominant litters. The two C57BL/6 mice that were not protected upon WT challenge were both male, raising the possibility of sex-based differences in the immune response—a phenomenon that is well documented in the context of other vaccines (21). These encouraging results suggest the potential for a single, low-dose vaccine to provide prolonged protection. However, it is possible that a higher vaccine dose or boosting could improve immunogenicity and subsequent protection. Taken together, this work provides key preliminary data that justify larger future studies that are sufficiently powered to detect sex- and dose-based differences in ΔNSsΔNSm immunogenicity and efficacy over longer durations.

Materials and methods can be found in the supplemental material.

## SUPPLEMENTAL MATERIAL

Supplemental material msphere.00894-25-s0001.docx Materials and methods and Figure S1.

ASM does not own the copyrights to Supplemental Material that may be linked to, or accessed through, an article. The authors have granted ASM a non-exclusive, world-wide license to publish the Supplemental Material files. Please contact the corresponding author directly for reuse.
