# Supplementary material for: Durable protection against lethal Rift Valley fever hepatitis and encephalitis following low-dose ΔNSsΔNSm vaccination in mice
Source: mSphere. 2026 Feb 27;11(3):e00894-25. doi: 10.1128/msphere.00894-25 (PMC13037407; doi:10.1128/msphere.00894-25)
Supplement: Supplemental material — Materials and methods and Figure S1. [file msphere.00894-25-s0001.docx]

**Supplemental information**

**Supplemental figure**


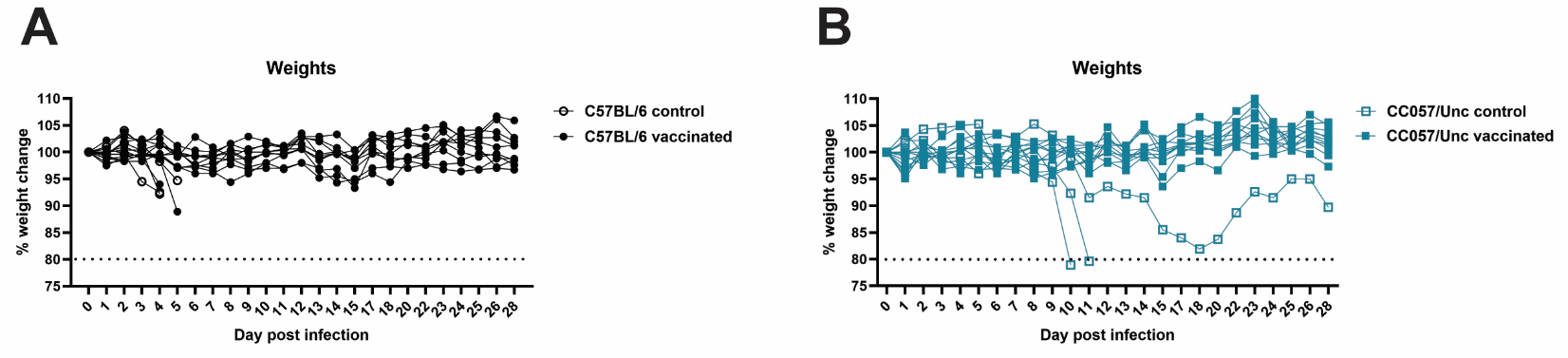


**SFigure1**: **Animal weights following WT RVFV challenge.** Individual weights are plotted as percent increase or decrease from starting weight (day 0) for (**A**) C57BL/6 and (**B**) CC057/Unc mice. The dotted line indicates a 20% weight loss, which is a euthanasia criteria.

**Materials and Methods**

**Biosafety**. All experiments with WT RVFV (ZH501) were conducted in Biosafety Level 3 (BSL-3) and Animal Biosafety Level 3 (ABSL-3) facilities at the University of Pittsburgh Regional Biocontainment Laboratory (RBL), which has Division of Select Agents and Toxins (DSAT) approval for work with Select Agents. Work with ΔNSsΔNSm was performed at BSL-2.

**Animal studies**. This study complied with institutional guidelines, and all procedures were approved by the University of Pittsburgh Institutional Animal Care and Use Committee (protocol 25036264).

**Mouse experiments**. A breeding trio consisting of two females and one male CC057/Unc mice were obtained from the UNC Collaborative Cross Resource and used to initiate and maintain an off-site breeding colony (Charles River). Female and male CC057/Unc (4 to 12 weeks old) were shipped from Charles River to the University of Pittsburgh. Female and male C57BL/6 mice (4 -12 weeks old) were purchased from the Jackson Laboratory (Strain #000664). Mice were housed within ABSL-2 facilities. Prior to WT RVFV challenge, mice were transferred to ABSL-3 laboratories and housed in microisolator cages in HEPA filtration racks, following standard barrier techniques.

Mock vaccination (sterile PBS, Gibco #10010), vaccination with attenuated RVFV, and challenge with WT RVFV (diluted in PBS from stocks to desired TCID_50_) were performed via left foot pad injection (20 μL total) under isoflurane anesthesia. All viruses were back-titered at the time of administration to confirm the dose given. Where applicable, blood was collected from mice at specified timepoints via lateral saphenous bleed into serum separator tubes and centrifuged for serum purification. Animals were checked daily and weighed weekly for up to 6-months post-vaccination. Animal groups and numbers are as follows: 20 TCID_50_ ΔNSsΔNSm (n=10 per mouse strain, 2 females and 8 males); mock vaccinated animals/ 2TCID_50_ WT RVFV (*n*=3 per mouse strain, 1 female and 2 males); 20 TCID_50_ ΔNSsΔNSm/ 2TCID_50_ WT RVFV (*n*=12 per mouse strain, 4 females and 8 males).

Mice were evaluated for clinical signs of disease once a day post WT RVFV challenge and euthanized according to a predetermined clinical illness scoring algorithm ^1^. At the time of euthanasia, mice were anesthetized with isoflurane (Piramal, #NDC 66794-017-10), bled via cardiac puncture for serum isolation and then euthanized through cervical dislocation. Tissues collected included liver, spleen and brain. For splenocyte isolation, spleens were collected in 5mL RPMI-1640 (Gibco, #A10491) with 10% FBS, and splenocytes were prepared using manual disruption ^2^.

**Virus strains and cell culture**. Stocks of recombinant WT RVFV (strain ZH501) and RVFV lacking viral proteins NSs and NSm (ΔNSsΔNSm) were produced using reverse genetics as previously described ^3^, grown to passage 2 and sequence confirmed via Illumina sequencing prior to use. The RVFV reverse genetics system was kindly shared by César Albariño of the US Centers for Disease Control and Prevention Viral Special Pathogens Branch. Vero E6 cells (ATCC CRL-1587) were maintained in Dulbecco’s Modified Eagle Medium (Gibco, #10566) with 10% fetal bovine serum (Gibco, #16000) and 1x Antibiotic-Antimycotic (Gibco, #15240) at 37°C, 5% CO_2_ using conventional cell culture techniques. Viral titers were determined as 50% tissue culture infective dose (TCID_50_) by serial dilution and incubation on Vero E6 cells ^1^. Plates were stained by indirect fluorescent-antibody assay (IFA) using a monoclonal mouse IgG1 anti-RVFV N primary antibody (custom, Genscript) and the Alexa Fluor^TM^ 488 goat anti-mouse IgG secondary antibody (Invitrogen, #A11001), and TCID_50_ was assessed using Reed & Muench ^4^.

**RNA extraction and RVFV qRT-PCR**. Tissues from *in vivo* experiments were collected into pre-weighed grinding vials (Fisher, #15-340-154) containing 500 μL PBS and 1x Antibiotic-Antimycotic and re-weighed prior to homogenization with the D2400 Homogenizer (Benchmark Scientific). RNA was extracted using TRIzol reagent (Invitrogen, #15596018) and the Direct-zol^TM^ MiniPrep Plus kit (Zymo Research, #R2072). Sample RNA was assayed by qRT-PCR for the RVFV L segment using Reliance One-Step Multiplex RT-qPCR Supermix (BioRad, #12010221) and viral RNA loads were determined based upon an RNA standard curve as previously described ^1^. The following conditions were used: 50°C for 15 minutes (min), 95°C for 3min, and then 40 cycles of 95°C for 15 seconds (s), and 55°C for 1min. The assay limit of detection (LOD) is set as the lowest amount of detectable standard curve RNA normalized to the average tissue weight.

**Enzyme-linked immunosorbent assay (ELISA)**. Lysates were generated from either uninfected Vero E6 cells or RVFV ΔNSsΔNSm infected Vero E6 cells. Briefly, cells were infected at a multiplicity of 1 and once infected cells reached >75% cytopathic effect, the cells from 10 T150 flasks were combined and resuspended in 5mL lysis solution (1% Triton X-100 in PBS with protease inhibitor (ThermoFisher, #A32963)). Lysates were sonicated for 10 min and clarified by centrifugation. MaxiSorp^TM^ plates (Fisher, #44-2404-21) were coated with lysates from either uninfected or ΔNSsΔNSm infected Vero E6 cells and incubated at 4°C overnight. For RVFV NSs-specific ELISA, plates were coated with SARS-CoV-2 N protein ^5^ as the negative control or recombinant RVFV NSs (custom, Genscript) at 200 ng of protein per well in sterile PBS and incubated at 4°C overnight. The following day, plates were incubated with blocking buffer (5% non-fat milk in PBS-Tween 0.1%, PBST) for 1 hour (h). In duplicate, mouse serum samples collected either pre-challenge or at the time of euthanasia, as well as a negative mouse control serum, were serially diluted in blocking buffer and incubated on plates for 2h at 37°C. Plates were then washed three times with PBST and incubated with HRP-conjugated donkey anti-mouse IgG (Jackson ImmunoResearch, #715-035-150 ) at 1: 5,000 in blocking buffer for 1h at 37°C. Plates were again washed three times with PBST and tetramethylbenzidine (TMB) substrate (SeraCare, #5120-0038, 5120-0049) and subsequently TMB stop solution (SeraCare, #5150-0021) were added to the plates. The optical density (OD) at 450nm was measured via Biotek Synergy plate reader. The endpoint titer was defined as the highest dilution of serum that resulted in an OD value at least three standard deviations above the average obtained from all negative mouse serum control wells.

**Focus reduction neutralization assay (FRNT)**. In duplicate, mouse sera were serially diluted in cell culture media and incubated with an equal volume of media containing 200 foci-forming units (FFUs) of ΔNSsΔNSm for 1h at 37°C. Vero E6 cells in 96-well format were subsequently incubated with the serum-virus mix for 1h at 37°C. The inoculum was then replaced with 1.5% carboxylmethylcellulose (CMC) (Sigma, #C4888)/ 1x Modified Eagle Medium (MEM), diluted from 3% CMC in 2x MEM (Gibco, #11935), for 18h. Plates were washed with PBS, fixed in 10% formalin for 20min, and again washed in PBS. Plates were then permeabilized with 0.1% Triton-X100 in PBS for 10min at room temperature (RT), washed with PBS, and blocked in 5% non-fat-milk in PBST for 1h. Immunostaining was performed using a rabbit polyclonal anti-RVFV N primary antibody at 1:1,000 (custom, Genscript) in 5% non-fat-milk in PBST for 1h at RT followed by a horseradish peroxidase (HRP)-conjugated donkey anti-rabbit secondary antibody at 1:1000 (Jackson Immuno Research, #711-035-152) for 1h at RT. Foci were visualized using TMB substrate (MossBio, #TMBH-1L). Plates were dried overnight and imaged using the CTL Immunospot reader. The focus reduction neutralization titer (FRNT_80_) was defined as the dilution of serum that neutralized 80% of input FFU.

**T cell ELISPOT**. To assess virus-specific T cells following vaccination, an IFN-γ mouse ELISPOT assay (Mabtech Inc., #3321-4HPT-2) was used following the manufacturer’s instruction and as previously described ^6^. Briefly, splenocytes were isolated using manual disruption and incubated on pre-coated MultiScreen® IP sterile plates (Millipore, #MSIPS4W10) with negative control (DMSO vehicle), positive control (Cell Activation Cocktail, PMA/Ionomycin, Biolegend, #423301) or peptide pools (2 μg/mL final concentration) that represent the entirety of each viral structural protein in 15mer peptides with 11mer overlaps. Peptides in each pool are as follows: RVFV nucleoprotein N (*n*=59), glycoprotein Gc ([Gc1, *n*=61], [Gc2, *n*=62]), and glycoprotein Gn ([Gn1, *n*=70], [Gn2, *n*=70]). Spots were counted on the CTL Immunospot reader. Data for Gn and Gc are reported as the combined value from the 2 respective peptide pools. Peptide pools for these proteins had to be divided into 2 pools to avoid DMSO toxicity.

**Flow cytometry.** A flow cytometric assay was also used to assess virus-specific T cells following vaccination. Splenocytes were washed in RPMI-1640 (Gibco, #A10491) with 10% FBS then incubated with either positive control (Cell Activation Cocktail, PMA/Ionomycin Biolegend, #423301), negative control (Dimethyl Sulfoxide, DMSO, Sigma, #D2650) or RVFV peptide pools (2 μg/mL final concentration) for 6h at 37°C in the presence of 10 μg/mL brefeldin A (Brefeldin A solution, Invitrogen, #50-112-9757). They were then washed in PBS, incubated in LIVE/DEAD near IR (Thermo Fisher, #L34976) at 1:500 for 10 min. Following a wash in flow buffer (PBS with 2% FBS) cells were stained for 30min using the following antibodies: BV510 CD3 (17A2, Biolegend, #100234), BUV395 CD4 (RM4-5, BD, #740208), PE/Cy7 CD8a (53-6.7, BD, #552877), BV421 CD44 (IM7, Biolegend, #103039), APC/Cy7 CD19 (6D5, Biolegend, #115530), APC/Cy7 CD14 (Sa14-2, Biolegend, #123318). Cells were then washed twice in flow buffer, and fixed with BD Cytofix/Cytoperm^TM^ (BD Biosciences, #51-2090KZ). After fixation and permeabilization, cells were washed in BD Perm/Wash (BD Biosciences, #51-2091KZ) and stained with Alexa647 IFN-γ (XMG1.2, BD, #557735) for 45min and then again washed prior to acquisition on an LSRII. Gating strategy: Lymphocytes were identified by forward and side scatter, then single cells were gated using forward scatter area versus height. A time gate was applied for homogeneity then live T cells were determined as CD3+/CD19-/CD14-/live-dead near IR negative. CD3+ cells were then separated into CD4+ and CD8+ populations. Of these populations, virus-specific T cells were defined as CD44+/IFN-gamma +. Data were analyzed using FlowJo. Data for Gn and Gc are reported as the combined value from the 2 respective peptide pools. Peptide pools for these proteins had to be divided into 2 pools to avoid DMSO toxicity.

**Data processing and statistical analysis.** All graphs were generated and statistical analyses performed using GraphPad Prism 10. Figures were generated using Adobe Illustrator and timelines within the figures were generated with BioRender.com.

**References**

1 Cartwright, H. N., Barbeau, D. J. & McElroy, A. K. Rift Valley Fever Virus Is Lethal in Different Inbred Mouse Strains Independent of Sex. *Front Microbiol* **11**, 1962 (2020). <https://doi.org/10.3389/fmicb.2020.01962>

2 Harmon, J. R. *et al.* CD4 T Cells, CD8 T Cells, and Monocytes Coordinate To Prevent Rift Valley Fever Virus Encephalitis. *J Virol* **92** (2018). <https://doi.org/10.1128/JVI.01270-18>

3 Gerrard, S. R., Bird, B. H., Albarino, C. G. & Nichol, S. T. The NSm proteins of Rift Valley fever virus are dispensable for maturation, replication and infection. *Virology* **359**, 459-465 (2007). <https://doi.org/10.1016/j.virol.2006.09.035>

4 Reed, L. J. M., H. A simple method of estimating fifty per cent endpoints. *Am J Epidemiol* **27**, 493-497 (1938). <https://doi.org/10.1093/oxfordjournals.aje.a118408>

5 Xu, L. *et al.* A Cross-Sectional Study of SARS-CoV-2 Seroprevalence between Fall 2020 and February 2021 in Allegheny County, Western Pennsylvania, USA. *Pathogens* **10** (2021). <https://doi.org/10.3390/pathogens10060710>

6 Barbeau, D. J. *et al.* Rift Valley Fever Virus Infection Causes Acute Encephalitis in the Ferret. *mSphere* **5** (2020). <https://doi.org/10.1128/mSphere.00798-20>
